# Microsatellite Instability in Colorectal Cancer: Time to Stop Hiding!

**DOI:** 10.18632/oncotarget.353

**Published:** 2011-11-12

**Authors:** Alex Duval, Ada Collura, Kevin Berthenet, Anaïs Lagrange, Carmen Garrido

**Affiliations:** ^1^ Institut National de la Santé et de la Recherche Médicale (INSERM), Centre de Recherche Saint-Antoine, Equipe ‘Instabilité des Microsatellites et Cancers’, Paris, France; ^2^ Université Pierre et Marie Curie Paris, France; ^3^ INSERM, Dijon, France; ^4^ University of Burgundy, Dijon, France; ^5^ CHU Dijon, France

Colorectal cancer (CRC) is the second cause of cancer-related death worldwide. Surgery constitutes the primary therapy for these tumors, together with chemotherapy that is usually recommended in patients with metastatic primary CRC. Although molecularly distinct entities arising from different physiopathogenic mechanisms - microsatellite (MSI) and chromosomal instability (also called microsatellite stable, MSS) - have been characterized in CRC, there is still no specific therapeutic approach that takes into account disease's molecular heterogeneity [1]. MSI is observed in 10-15% of sporadic CRCs. MSI CRCs displayed particular morphologic features, with greater predilection for the right colon, mucinous histology, low metastatic power, poorer differentiation and higher numbers of tumor-infiltrating lymphocytes. They have been consistently reported to show an improved prognosis and a different response to chemotherapeutic agents. In a recent article in *Nature Medicine*, we have reported the specific mutation of the molecular chaperone HSP110 in MSI CRCs and how the presence of this mutant may constitute a first step towards the understanding of their particular clinical characteristics [2].

It is now well established that MMR deficiency is not in itself a direct transforming event and that the development of these tumors is MSI-driven. This distinctive MSI pathway is characterized by somatic mutational events affecting short coding repeated sequences that, when having an oncogenic effect, provide selective pressure during tumor progression [3]. We showed that a T_17_ mononucleotide repeat located in intron 8 of *HSP110* was systematically mutated in MSI CRC cell lines and primary tumors [2]. The shortening of this repeat in tumor DNA correlated with increased synthesis of an aberrant *HSP110* transcript due to exon 9 skipping, to the detriment of wild-type *HSP110* mRNA. As a result, a truncated HSP110 mutant protein (HSP110DE9) accumulated in MSI tumors. Strikingly, we demonstrated that HSP110DE9 acts as a dominant negative mutant that binds to HSP110 abrogating its chaperone activity and cytoprotective function. In colon tumors, HSPs including HSP110 have been clearly shown to promote cancer cell survival, protecting oncogenic proteins and inhibiting apoptosis [4-6]. It is thus unclear why HSP110DE9 proapoptotic mutant is selected during MSI tumorigenesis. Long, noncoding mononucleotide repeats such as the T_17_ located in *HSP110* intron 8 constitute hot spots for mutations in MSI tumors due to the MMR deficiency. Our hypothesis is that when these mutations are endowed with a biological anti-cancer activity, as it is the case with HSP110DE9, they can represent an Achilles' heel in the MSI-driven tumorigenic process. Further studies are now necessary to determine the exact role of HSP110DE9 during MSI tumor progression and to understand the contribution of HSP110DE9 in the more favorable prognosis of CRC MSI compared to MSS patients.

*In vitro*, HSP110DE9 expression sensitized colon cancer cells to anticancer agents such as oxaliplatin and 5-fluorouracil, which are routinely prescribed in the adjuvant treatment of patients with CRC [7]. In line with these results, we observed that MSI CRC patients with high HSP110DE9 expression levels who received chemotherapy were all associated with disease-free survival [2]. Therefore, HSP110DE9 levels are likely to constitute a crucial determinant for MSI CRC patients' prognosis and treatment response. Because this mutant protein was expressed at variable levels in these tumors, our findings thus provide evidence for an additional layer of clinical heterogeneity among MSI colon cancers. Additional studies in larger populations are now being performed in order to confirm these results. MSI CRC patients have been recurrently reported to benefit less from 5-FU treatment whereas they seem to show improved response to 5-FU-oxaliplatin (FOLFOX) that constitute today the gold standard of adjuvant chemotherapy in CRC. As Dr. Andrew T. Chan mentions in a recent issue in *Nature Medicine* [8], *“it is fascinating to speculate that such studies might show a lack of response to 5-FU confined to the MSI CRCs with low levels of HSP110DE9”*.

In tumor samples, MSI phenotype can be determined by PCR according to international criteria or by immunohistochemistry studying mismatch repair (MMR) protein expression affecting MLH1, MSH2, MSH6 or PMS2. Our findings highlight that routine evaluation of the MSI phenotype together with investigation of HSP110 status could be of clinical interest in CRC diagnosis. Note worthily, HSP110DE9 is the first HSP mutant identified in a cancer so far. Developing inhibitors of HSP110 that mimic the anti-cancer chemosensitizing effect of HSP110DE9 is also a promising perspective.
